# Definition, measurement, and function of pore structure dimensions of bioengineered porous bone tissue materials based on additive manufacturing: A review

**DOI:** 10.3389/fbioe.2022.1081548

**Published:** 2023-01-04

**Authors:** Wen Peng, Yami Liu, Cheng Wang

**Affiliations:** ^1^ Department of Orthopaedic Surgery, The First Affiliated Hospital, Hengyang Medical School, University of South China, Hengyang, China; ^2^ Foshan Orthopedic Implant (Stable) Engineering Technology Research Center, Foshan, China

**Keywords:** additive manufacturing, bioengineered materials, bone tissue, pore structure, dimension characterization

## Abstract

Bioengineered porous bone tissue materials based on additive manufacturing technology have gradually become a research hotspot in bone tissue-related bioengineering. Research on structural design, preparation and processing processes, and performance optimization has been carried out for this material, and further industrial translation and clinical applications have been implemented. However, based on previous studies, there is controversy in the academic community about characterizing the pore structure dimensions of porous materials, with problems in the definition logic and measurement method for specific parameters. In addition, there are significant differences in the specific morphological and functional concepts for the pore structure due to differences in defining the dimensional characterization parameters of the pore structure, leading to some conflicts in perceptions and discussions among researchers. To further clarify the definitions, measurements, and dimensional parameters of porous structures in bioengineered bone materials, this literature review analyzes different dimensional characterization parameters of pore structures of porous materials to provide a theoretical basis for unified definitions and the standardized use of parameters.

## Introduction

### Technical background

Repair of bone tissue defects caused by tumors, infections, trauma, and medically induced injuries are the main applications of bioengineered porous bone tissue materials. The ability of these materials to promote bone tissue repair and reconstruction has been widely recognized (Xia et al., 2023; Dall'Ava et al., 2020; Chen et al., 2018). The bioengineered porous bone tissue materials promote bone tissue reconstruction and repair by providing an effective support effect and maintaining a good mechanical environment at the defect site after implantation (Ponader et al., 2010; Wang et al., 2020; Lehder et al., 2021). They also act as a scaffold for tissue growth, enabling the growth and formation of fibers, blood vessels, and bone tissue (Harrison et al., 2014; Taniguchi et al., 2016; Wu et al., 2022). Moreover, due to their unique porous structure, the overall elastic modulus of the implants can be effectively reduced to avoid stress shielding and provide the necessary stress stimulation for bone tissue growth (Shah et al., 2016; Cheong et al., 2018; Jette et al., 2018; Chao et al., 2021). In addition, such materials can promote the integration of the tissue‒scaffold interface by constructing special morphologies on the material surface (Stevenson et al., 2016; Shuai et al., 2018; Kopp et al., 2019). They can also accelerate the bone defect repair and reconstruction process by relying on active substances, such as bone growth factors, that contribute to the formation of bone tissue (Li et al., 2015; Li et al., 2019a; Huang et al., 2020). They can also be prepared as slowly degradable porous materials using degradable metals, polymers, or other biomaterials, *etc.*, eventually leading to complete bone tissue replacement (Carluccio et al., 2020; Cockerill et al., 2020; Wu et al., 2021; Qin et al., 2022a; Qin et al., 2022b). At present, such bioengineered porous bone materials constructed by additive manufacturing technology have replaced traditional techniques such as the direct foaming method, pore-forming agent method, and powder sintering method due to their controlled pore structure, reliable mechanical properties, and flexible formulation mechanism (Zaharin et al., 2018; Cao et al., 2020; Sharma et al., 2020).

### Structure design

Currently, the pore structure design used for bioengineered porous bone tissue materials mainly includes two approaches: regular and irregular pore structures. In this line, there are two main technical solutions to constructing a regular pore structure: through the regular arrangement of rod structures in 2D planes and through the superposition of 2D planes in the *Z* axis (Cubo-Mateo and Rodriguez-Lorenzo, 2020; Sakthiabirami et al., 2021; Kilian et al., 2022). On the other hand, rod structures form the unit cells in 3D spaces through angular connections, and then the whole structure is formed by stacking the unit cells in the X/Y/*Z* axes (Li et al., 2018a; Li et al., 2018b). In recent years, the pore structure based on triply periodic minimal surface (TPMS) has emerged. It mainly involves the periodic extension of parametric surface sheets in three directions, culminating in the formation of walls in a 3D space state and a complete pore structure (Kelly et al., 2019; Corona-Castuera et al., 2021; Lu et al., 2022a; Lv et al., 2022). However, the design of irregular pore structures is more complex. It requires CT scanning of bone tissue to obtain the 3D structure of bone trabeculae, which are then rotated, stitched, and superimposed to achieve the overall design of the pore structure (Cheng et al., 2014). Alternatively, by employing Voronoi structures, it generates randomly distributed points within the spatial structure under certain conditions in a functional manner, subsequently forming pore structures with unspecified arrangements by connecting points to points (Deering et al., 2021; Zhao et al., 2021; Zhu et al., 2021). In addition to the above structures, the gradient pore structure has gradually become a research hotspot in recent years as the knowledge of the pore structure of bioengineered porous bone tissue materials has gradually deepened. These structures are usually implemented by parameterized adjustments of the pore structure sizes (Li et al., 2020a; Kamboj et al., 2020), rod diameters (Li et al., 2019b; Zhang et al., 2019; Li et al., 2020b; Liu et al., 2020; Xiong et al., 2020), or wall thicknesses (Afshar et al., 2016), changing the random distribution conditions of the point arrangement (Zhao et al., 2021; Zhu et al., 2021), or directly splicing different pore structures (Wieding et al., 2014; Wysocki et al., 2016; Kayacan et al., 2018) based on conventional pore structures.

## Current problems

**FIGURE 1:**
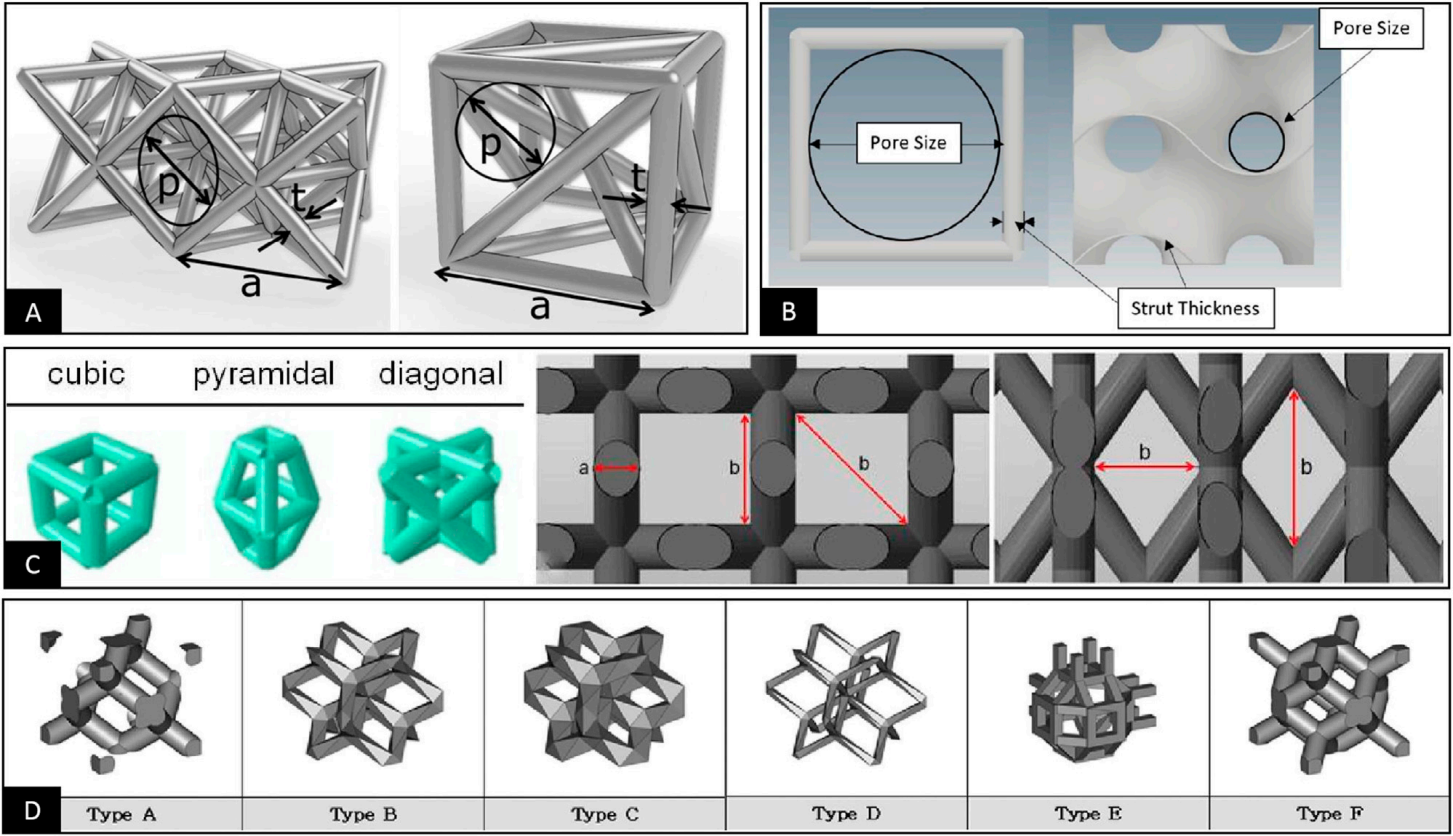
**(A)** The pore size is used to characterize the dimensional parameters of the pore structure. The pore sizes of different pore structures are defined using the maximum inner tangent circle in three dimensions and the maximum inner tangent circle in two dimensions, respectively (Arabnejad et al., 2016). **(B)** The pore size is used to characterize the dimensional parameters of the pore structure, and both define the maximum inner tangent circle of the 2D plane within the pore structure as the pore size. However, there are significant differences in the positioning of the pore size (Zaharin et al., 2018). **(C)** The pore size is used to characterize the dimensional parameters of the pore structure. The pore size is respectively measured in length and width and diagonally for the three different structures (Markhoff et al., 2015). **(D)** The unit cell size is used to characterize the dimensional parameters of the pore structure. Two different definitions of the unit cell are used for the six different pore structures (Lim et al., 2017).

To characterize the pore structure dimension of porous materials, the pore size is often used as an important parameter and as the main control index to study the mechanical and biological properties of porous materials. However, after summarizing the relevant literature, we found that the definition of pore size is not clear in some literature sources. Furthermore, no unified positioning and measurement methods are available (Yavari et al., 2013; Amin Yavari et al., 2014; Soro et al., 2019; Liu et al., 2022). This problem has prompted some studies to cite findings from other literature with significant differences in the definition, localization, and measurement of pore size, leading to questioning the conclusions in their papers (Zadpoor, 2015; Dziaduszewska and Zielinski, 2021). More importantly, some studies have even used different definitions and positioning methods of pore sizes to describe different pore structures, possibly leading to some bias in comparative analyses of different structures, as shown in Figures 1A–C–C (Markhoff et al., 2015; Arabnejad et al., 2016; Zaharin et al., 2018; Hossein Ehsani et al., 2022). Probably due to this uncertainty regarding the definition of pore size, some studies have abandoned using pore size for evaluating pore structure and have instead used the unit cell size as a measure to characterize pore structure dimension in porous materials (Yan et al., 2015; Hedayati et al., 2017; Lim et al., 2017; Ahmadi et al., 2019; Lu et al., 2020; Yang et al., 2020). However, differences in the definition of the unit cell size ultimately lead to the inconsistent characterization of the pore structure size, as shown in Figure 1D (Lim et al., 2017). Considering the current confusing situation, we analyze and summarize the definition and measurements of the pore structure dimensions. In addition, the specific mechanisms affecting osteogenesis and bioengineered porous bone tissue materials are evaluated to clarify definitions and facilitate further material research and industrial applications based on previous research.

## Definition of pore structure dimension

### Conventional porous materials

Early in the field of bone tissue bioengineering, porous materials were mostly made by processes such as vapor-phase porogenesis (Oppenheimer and Dunand, 2010; Ji et al., 2012), blowing agent porogenesis (Kato et al., 2013; Kapat et al., 2017; Liu et al., 2017), and solid-phase porogenesis (Maya et al., 2012; Hsu et al., 2013; Yamanoglu et al., 2016), in which the porosity of porous materials can usually be precisely controlled by the volume of added porogenic agents or binders. However, the morphology of the internal pore structure and the pore size of porous materials cannot be effectively controlled. Considering the irregular pore shape, uneven distribution, and easy formation of closed pores in porous materials, technicians use spherical porogenic agents filled with regular particles and controlled particle size to form a uniformly distributed and regular morphological pore structure in porous materials (Jia et al., 2015). The preparation of porous materials by controlled particle porogenic agents enables the precise control of the morphology of the pore structure within porous materials, further making the pore size in the pore structure an important parameter to influence the mechanical and biological properties of these materials (Imwinkelried, 2007; Zhao et al., 2016). This molding technique involves mixing the granular material with the target material, casting, molding, and then removing the granular material by solution elution or high-temperature sintering after the material is formed to finally form a porous structure within the material with the outline of a granular material (Ye and Dunand, 2010). From the summary analysis, we concluded that the pore diameter in this type of porous material should represent the size of the 3D space inside the pore structure of the material, which is used to evaluate the maximum size to which the cells within the pore structure can grow (Cao et al., 2020). The pore throat size represents the size of the 2D planar channel used to achieve communication between adjacent pore structures on the surface of porous materials. It is used to evaluate the maximum planar size of the pore structure that can accommodate cells growing into the pore structure (Otsuki et al., 2006). Although the shape of porous materials produced by the conventional process can only achieve a simple geometry, this defect restricts the industrial application of these materials in bone tissue engineering. However, this method to characterize the pore structure dimension using both pore size and pore throat size is straightforward and should be used as an important reference and guide for defining pore structure dimensions.

### Additive manufacturing of porous materials

The application of material-extrusion-based 3D printing (ME-3DP), such as fused deposition modeling (FDM) (Diez-Escudero et al., 2020) and direct ink writing (DIW) (Lewis, 2006) in the preparation of bioengineered porous bone tissue materials has, to some extent, solved the problems of inability to achieve a specific shape and inaccurate connectivity of the pore structure of porous materials prepared by conventional processes (Li et al., 2021). This technique is mainly used to extrude solid or slurry materials in 2D planes according to a specific scanning path and form a planar structure with pore morphology. It is followed by superimposing multiple planar structures with a certain thickness in the *Z* axis sequentially to finally build pore structures of porous materials (Cubo-Mateo and Rodriguez-Lorenzo, 2020; Sakthiabirami et al., 2021; Yang et al., 2021; Kilian et al., 2022). Considering this molding technology’s characteristics, the prepared material’s structure in the 2D plane can be controlled. Thus, its structure definition in the 2D plane is also accurate, but the 3D space structure cannot be accurately defined and positioned because the layer thickness cannot be precisely controlled. Therefore, the literature continues to use pore size as a specific parameter to define the pore structure dimension, defining it as the spacing of two parallel rods in a 2D plane (Shanjani et al., 2017; Gupta et al., 2021). However, some literature ignores the rod diameter size and uses the scan spacing directly as the pore size (Kilian et al., 2022). Nevertheless, according to the original definition of pore size and pore throat size, such parameters should be defined as the pore throat size in the 2D plane, not pore size in 3D space. The fundamental reason for this discrepancy is the objective drawback of the preparation technique of insufficient strength in molding and accuracy after molding.

With further developments in additive manufacturing technology, preparation techniques such as DLP (Schmidleithner et al., 2019), SLM (Yang et al., 2018; Pei et al., 2020), or EBM (Nune et al., 2017a; Zhang et al., 2020) have been gradually applied to bioengineered porous bone tissue materials, benefiting from the high-precision material formation of light-cured materials or powder materials by high-precision light sources or energy beams. In this context, the most typical and widely used technology is SLM, which mainly uses a micron laser beam to melt the micron powder material with high precision. Then the 2D plane structure is prepared by the movement of the laser beam, and the overall target structure is finally prepared by layer-by-layer processing (Wei et al., 2017). The advantages of these techniques over ME-3DP are the improved precision processing accuracy and the ability to build porous structures with controlled diameter, length, and tilt angle support structures in the *Z* axis through the individual superposition of 2D point structures in the *Z* axis. This results in a complete 3D space morphology in the porous structures; thus, most printed porous structures have controlled, regular, and connected 3D space shapes (Li et al., 2018a; Li et al., 2018b; Li et al., 2020c). Furthermore, as porous materials are prepared by relying on this molding technology, the control of various parameters of the structure during the molding process is more accurate, contributing to the appearance of different pore structure dimension definitions in the same type of structure. We found two definitions of pore diameter that follow the conventional molding process and ME-3DP, respectively, where the maximum internal tangent spherical diameter in the 3D space of the pore structure is taken as the pore diameter (Ambu and Morabito, 2019). In addition, the maximum internal tangent circular diameter in the 2D plane of the pore structure is defined as the pore diameter (Wauthle et al., 2015; Liang et al., 2022) in the relevant literature sources. According to the original definition of pore size and pore throat size, these two structures should be defined as pore size and pore throat size. Therefore, we believe this difference is the source of the current confusion in the academic community about the definition of the pore structure dimension of porous materials.

### Recommended definition method

**FIGURE 2:**
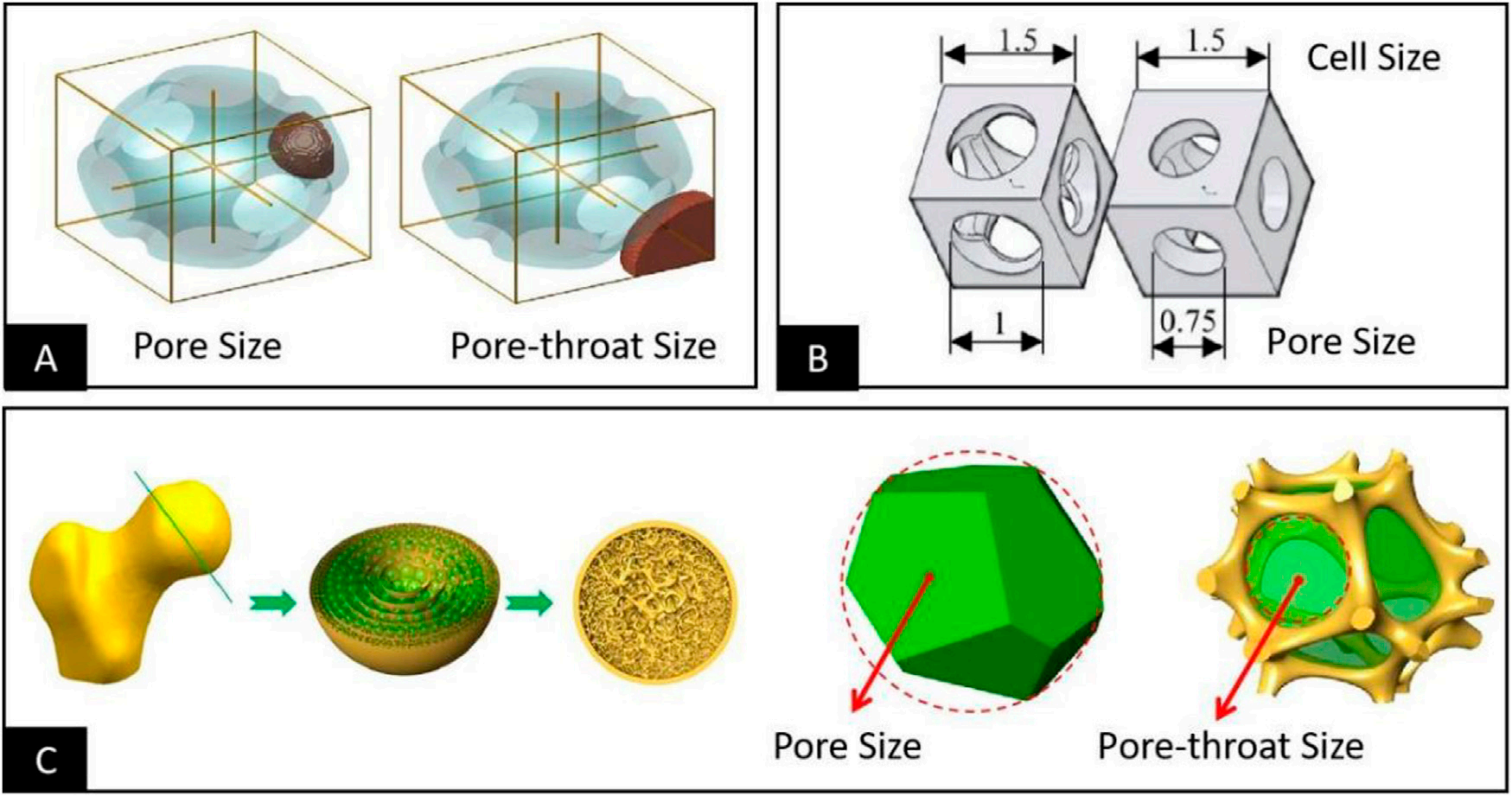
**(A)** Defining the maximum 3D space dimension that can be accommodated within the pore structure as the pore size and the maximum 2D plane dimension to interconnect the pore structure with other adjacent pore structures in the vicinity of the pore throat size (Afshar et al., 2016). **(B)** Defining the maximum 3D space dimension that can be accommodated within the pore structure as the unit cell size and the maximum 2D plane dimension to interconnect the pore structure with other adjacent structures in the vicinity as the pore size (Liverani et al., 2021). **(C)** Considering the structural morphology of human bone tissue, the bone trabecular structure consists of a large number of pore structures of different sizes; the pore size should be defined as the size of the 3D space structure in which the cells grow, and the pore throat diameter should be defined as the size of the 2D plane structure that allows the cells to be accommodated within the pore structure (Wang et al., 2018a).

Two different ways of defining the pore structure dimensions and specific schemes were finally summarized by analyzing the relevant studies involving bioengineered porous bone tissue materials in recent years. The first one defines the maximum 3D space dimension that can be accommodated within the smallest pore structure within the porous material as the pore size. Then it defines the maximum 2D plane dimension that interconnects the pore structure inside the porous material with other adjacent pore structures as the pore throat size, as shown in Figure 2A (Afshar et al., 2016; Jette et al., 2018; Ambu and Morabito, 2019; Wang et al., 2020; Lehder et al., 2021; Timercan et al., 2021). The second one defines the maximum 2D plane dimension of the pore within the porous material that interconnects with other adjacent pore structures as the pore diameter. It also introduces the unit cell size that contains a minimum pore structure and can be accumulated by repetition to form a complete porous material for measuring the maximum 3D spatial dimension within the pore structure, as shown in Figure 2B (Nune et al., 2017b; Melancon et al., 2017; Dallago et al., 2018; Barba et al., 2019; Liverani et al., 2021). These two methods of definition do not contradict each other in a practical sense; both describe the pore structure in 3D space and 2D plane simultaneously in different ways, and both meet the basic requirements for the pore structure dimensional characterization. However, the basic morphology of the cell is still different from that of the pore structure. Therefore, it usually contains some structures attributed to other adjacent pore structures, leading to the problem that the unit cell size is larger than the actual value when used to describe the maximum space for cell growth within the porous structure. Based on the above reasons and combined with the basic characteristics of human bone trabeculae (Wang et al., 2018a), as shown in Figure 2C, the bone trabecular structure is composed of many pore structures. However, their pore size and distribution follow stress stimulation. The pore size in the trabecular structure should be defined as the size of the 3D spatial structure in which cells, tissues, and tissue fluids grow, while the pore throat diameter should be defined as the size of the 2D planar structure that allows cells, tissues, and tissue fluids to enter the porous structure. We believe that adopting the first definition scheme is more consistent with the practical needs of bioengineered porous bone tissue materials to define and describe pore structure.

## Measurement of pore structure dimensions

### Positioning method

As analyzed previously, characterizing the 3D space shape of simple, rod, interleaved structures prepared by ME-3DP is difficult due to the inability to control the *Z* axis effectively. Therefore, to further position the pore size, most of the measurements in such porous materials are performed only for the pore throat size in the 2D plane (Lee et al., 2018). The rod lengths and thicknesses usually exhibit relatively disparate differences during the pore throat diameter measurement of this porous structure. Therefore, usually, two different sizes of pore throats are characterized separately in the horizontal and vertical planes, with the horizontal pore throat consisting of the lengths of interwoven rods in the *X* and *Y* axes and the vertical pore throat consisting of the thickness and length of the rod (Shanjani et al., 2017; Cubo-Mateo and Rodriguez-Lorenzo, 2020; Diaz-Gomez et al., 2020). Therefore, it is accurate to describe the pore throat size simply by using one of the parameter dimensions of length and width when the pore throat shape is described as a square (Hossein Ehsani et al., 2022). However, when its pore throat shape is described as a rectangle, its length and width should be reported separately, and the pore throat size should be expressed as length*width (Lee et al., 2018). However, considering the possibility of collapse and deformation of the material during the preparation process, which leads to an irregular shape of the hole throat (Baptista and Guedes, 2021), it is recommended to evaluate the pore throat size using the inner tangent circle diameter or equivalent circle diameter for such structures.

**FIGURE 3:**
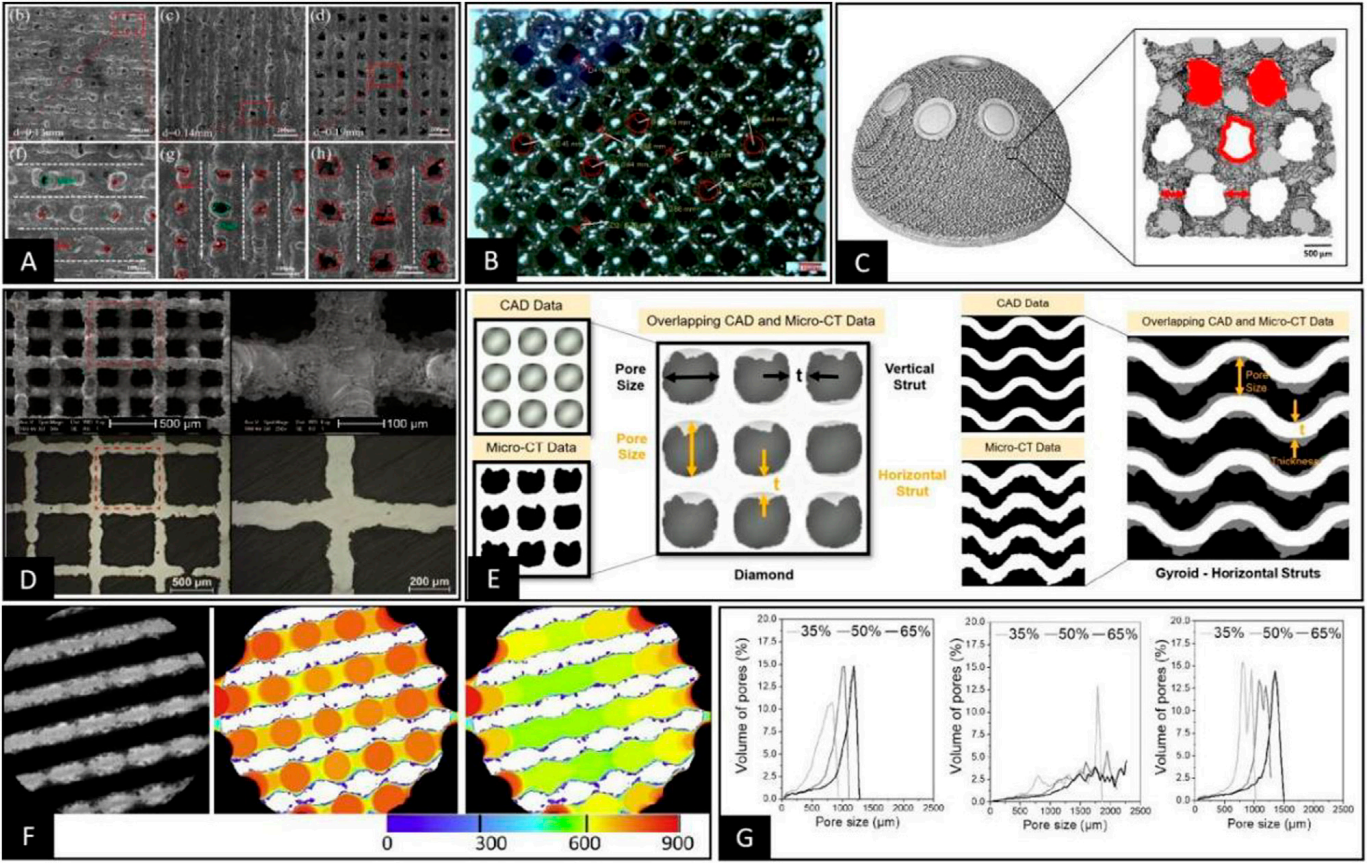
**(A)** Direct measurement of the inner tangent circle diameter of circular pore throats on the pore structure of porous materials prepared using SLM was performed under SEM (Lei et al., 2021). **(B)** Pore throat size was characterized by the 2D plane transformation of 3D space pore throats on the pore structure of porous materials prepared using EBM under a stereomicroscope, followed by measurement of the inner tangent circle diameter on the 2D plane (Moiduddin, 2018). **(C)** The 3D model of the pore structure of the porous material was obtained by Micro-CT, and a specific plane representing the pore throat size was selected. Then the pore throat size was measured by calculating the equivalent circle diameter of the pore throat (Dall'Ava et al., 2020). **(D)** The 3D pore structure was transformed into 2D planes using sample cutting, and the pore size data were measured indirectly by metallographic microscopy in 2D planes (Wauthle et al., 2015). **(E)** Representative cross-sections were obtained by Micro-CT, which adequately represent the pore morphology and measurements of the distance between two points or lines that are representative of the pore size within the 2D cross-section (Naghavi et al., 2022). **(F,G)** Using Micro-CT to obtain 2D cross-sectional images or 3D stereoscopic models of porous materials, the filling of different sizes of circular or spherical bodies was performed, as in Figure F (Taniguchi et al., 2016). Then the pore size distribution curves were plotted according to the size and number of different sizes of circular or spherical bodies, as in Figure G (Diez-Escudero et al., 2020).

In contrast, pore structures made by high-precision additive manufacturing technologies, such as SLM and EBM, have controllable dimensions in the X, Y, and *Z* axes and form a clear 3D space within the porous structure. This allows the pore size and the pore throat size of such structures to be clearly distinguished and located. For example, when the pore throat has a 2D plane geometry, such as octahedron type, in the measurement process, it is only necessary to select the 2D plane where the pore throat is located to measure the inner tangent circle of the pore throat, as shown in Figure 3A (Lei et al., 2021). However, when the pore structure is diamond-shaped, the quadrilateral structure forming the pore throat is distributed in 3D space. Therefore, the vertical direction of the maximum projected area of the quadrilateral structure should be selected to observe the pore throat size as a method to convert the 3D space to a 2D plane and then measure the inner tangent circle of the pore throat, as shown in Figures 3B,C (Dall'Ava et al., 2020; Moiduddin, 2018). However, when the pore throat is not a simple 2D structure or a 3D structure that can be planarly transformed, such as the TPMS-G type where the pore throat behaves as a spiral 3D channel, the results obtained by measuring the pore throat size only from a 2D plane do not necessarily match the actual situation (Zaharin et al., 2018; Wang et al., 2021; Su et al., 2022). Considering the gradual deepening of pore structure research, the complex shape of the pore structure will continue to be clarified. However, the complex 3D shape of the pore throat for accurately positioning the size poses a significant challenge, pending further research.

However, pore size, as a 3D space parameter, can be defined, located, and measured only when the 3D space form of the pore structure is complete and specific. Based on the localization methods for pore throat size summarized in the previous section, it is currently relatively difficult to perform pore size localization under 3D space conditions using a direct microscopic view. However, since the pore in the 3D state is the same as the pore throat in the 3D state, the transformation of the 2D plane can be performed by changing the observation angle and cutting the material when the pore structure is regular, as shown in Figure 3D (Wauthle et al., 2015; Zhang et al., 2020). However, in the 3D state, the pore has a more complex morphological structure compared to the pore throat, and sometimes it is only used to measure the pore size by considering the distance between two rods or walls that can roughly represent the pore size (Gorgin Karaji et al., 2017; Ma et al., 2019; Naghavi et al., 2022). Nevertheless, there are some special structures, such as TPMS-Split *p*, lidinoid types, and bionic trabecular structures, where it is impossible to specify the morphology of the aperture, making it impossible to determine the specific dimensions from 2D or 3D morphology based on the relative relationship between points, lines, and surfaces (Wang et al., 2018a; Zhao et al., 2021; Zhu et al., 2021). Therefore, in this situation, it is necessary to introduce the technical means in the 3D space state to measure the pore size directly based on the definition of pore size for the dimensions of the tangential spheres within the pore structure. This approach reduces the human bias in selecting 2D cross-sections and viewing the orientation; however, the accuracy of measurements in this technique depends heavily on the accuracy of Micro-CT scans and data processing.

### Measurement technology

The oldest pore size detection methods mostly started in the chemical and physical fields, including Mercury intrusion porosimetry (Zhang et al., 2013; Jiao et al., 2020). Although they can obtain pore size data more accurately, they are not suitable for detecting bioengineered bone materials because they are invasive methods and may be associated with other biological risks. In addition, since the core purpose of bioengineered bone materials is to realize the industrialization and clinical application of these materials, their detection method should be non-destructive, rapid, and accurate. Current methods for detecting pore size or pore throat size in bioengineered bone tissue materials include direct measurements performed by optical instruments such as SEM (Shuai et al., 2018; Lei et al., 2021) and indirect measurements relying on scanning devices such as Micro-CT (Cheng et al., 2014; Li et al., 2018a; Li et al., 2020c). Optical measuring instruments are, in essence, only a direct way of measuring in 2D plane conditions because their images cannot perceive the depth of the 3D space. Therefore, these techniques are only suitable for measuring 2D plane dimensions or 3D space dimensions that can be transformed through 2D planes (Wauthle et al., 2015; Shuai et al., 2018; Lei et al., 2021; Wu et al., 2021). This measurement method also has unique advantages. For example, the accuracy of direct measurement by optical instruments is significantly higher than that of Micro-CT-based 2D and 3D imaging measurements when the measurement target and evaluation method are specified. However, with the gradual advancement of pore structure-related research, the pore structure design is becoming increasingly complex. With the application of bionic non-regular pore structure and more different kinds of TPMS, the method used to measure pore structure dimension parameters, such as pore size or pore throat size on a 2D plane relying solely on optical instruments, is gradually replaced by other techniques.

Optical instruments are limited to 2D plane measurements in direct view and require destructive methods such as cutting or polishing. However, if planar switching of internal spatial structures is required, a highly accurate layer-by-layer scanning method of materials, such as Micro-CT or industrial CT, allows the acquisition of tomographic images for characterizing the layered morphology of materials and measuring local planes on tomographic 2D images (Yavari et al., 2013; Li et al., 2018a; Li et al., 2018b; Schmidleithner et al., 2019; Li et al., 2020c). The approach is, in essence, similar to that of direct measurement by optical instruments in the 2D plane, where the actual data measurement process is still highly dependent on the selection of 2D cross-section and view orientation, but its selection for 2D images are more accessible and accurate, as shown in Figure 3E (Otsuki et al., 2006; Dong et al., 2020; Naghavi et al., 2022). Because of the limitations of this technical solution in terms of subjective judgment, the aperture diameter is measured using the maximal covering spheres (MCS) method after the 3D reconstruction of images based on techniques such as Micro-CT. This automatic measurement technique works with spheres of different diameters by moving and filling the space structure until the boundary conditions are reached after the spheres form a tangent to the structure. Then the diameters of the spheres are included in the statistics and counted, eventually resulting in aperture diameter distribution curves related to the diameter and number of spheres, as shown in Figure 3F (Jones et al., 2007; Taniguchi et al., 2016; Timercan et al., 2021). In this measurement, the definition of pore size and pore throat diameter is ignored. Therefore, in the pore size distribution curves, we can observe a single pore structure, usually with a double or multiple peak pattern, respectively representing pore size and pore throat size, as shown in Figure 3G (Diez-Escudero et al., 2020). Another comparable technique is automatically measuring the trabecular separation (Tb.Sp) from Micro-CT 3D-reconstructed post data by software and using it to measure the pore size (Li et al., 2019b). Both of the above approaches are purely based on a comprehensive test of the 3D space size or distance, reflecting the approximate size of the pore and pore throat path in the form of average values and distribution curves. This type of measurement can accomplish pore size measurements for complex pore structures such as bone tissue trabecular structures, irregular structures, TPMS, gradient structures, and other porous scaffolds (Cheng et al., 2014; Wang et al., 2018a; Corona-Castuera et al., 2021; Zhu et al., 2021). However, this is more consistent with our specific needs for evaluating porous structures since the underlying logic of MCS in its measurement approach simulates the maximum size of cells that can pass and grow within the pore structure. In addition, clear distribution curves of pore and pore throat size can be obtained. Based on the above study, we believe that in the early development stage of porous structures and actual product quality sampling, techniques such as Micro-CT should be used to describe the pore size and pore throat size distribution curves. However, in the subsequent product treatment control process, optical instruments should be used for rapid and low-cost measurement of some of these structures that are easy to observe directly.

## Significance of pore structure dimension

### Function of pore throat size

**FIGURE 4:**
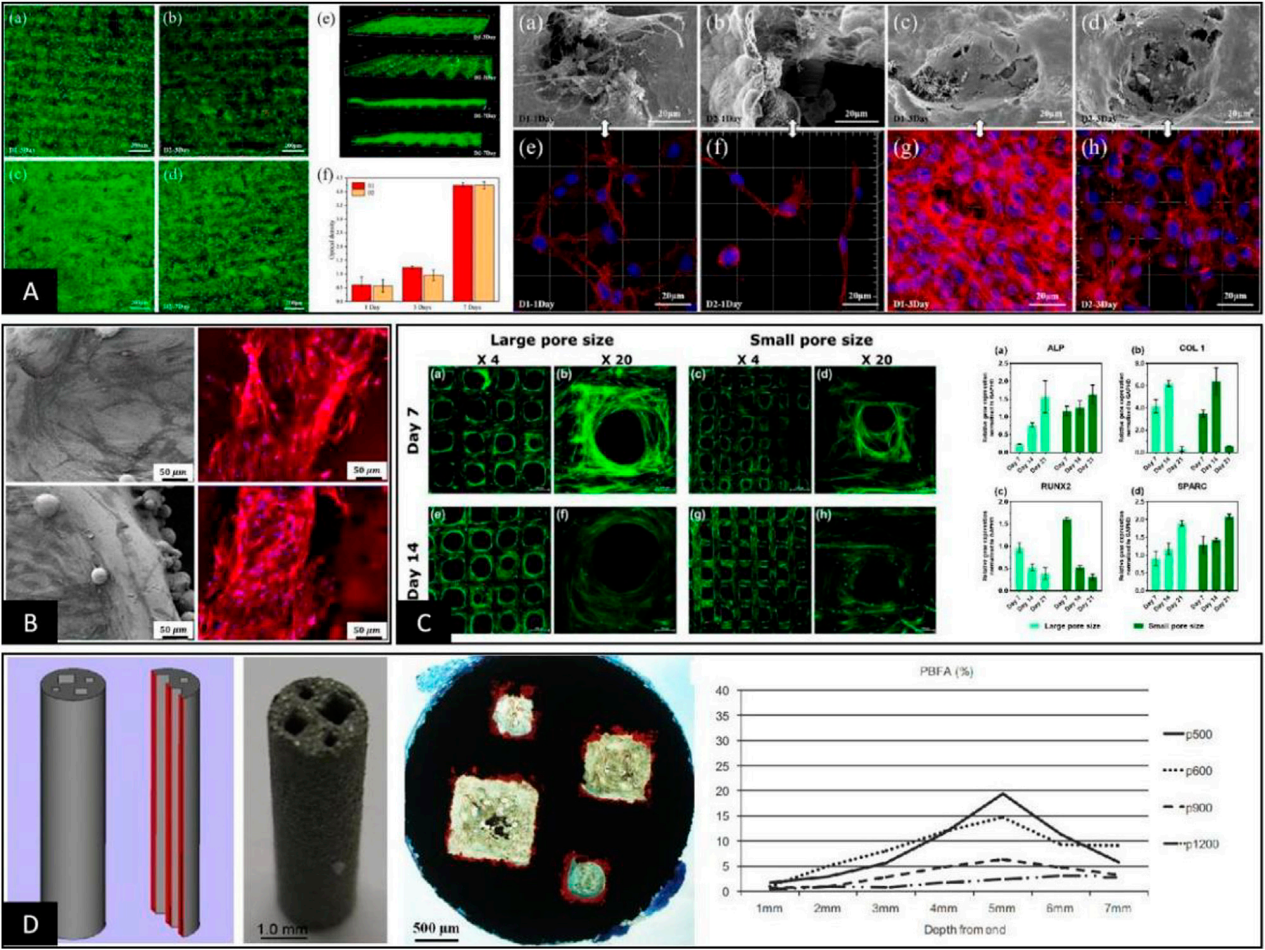
**(A)** When the pore throat sizes were 33 μm and 81 μm, the cells inoculated on the surface of the scaffold grew across the top of the pore structure and blocked the surface pore structure so that other cells could not enter the pore structure (Lei et al., 2021). **(B)** When the pore throat diameter was >100 μm, the cells grew on the internal rod or wall surface of the pore structure, and the pore throat of pore structure was not blocked by cell coverage, maintaining a good environment for nutrient exchange (Liu et al., 2020). **(C)** When the pore throat size met the basic requirements for cell growth into the pore, the smaller the pore throat size was, the better the state of cell adhesion, proliferation, and differentiation into bone, the mechanism of which may be related to the morphological differences in cell growth on the rod (Yang et al., 2021). **(D)** Cubic columnar pores with different pore throat sizes were significantly different in the osteogenic area within each pore during *in vivo* animal experiments, and the area of bone tissue varied depending on the distance from the end surfaces (Fukuda et al., 2011).

Since the relationship between pore throat size and pore size is determined by the shape of the pore structure, controlling changes in pore throat size usually results in changes in parameters such as pore size, porosity, and elastic modulus. Therefore, there are reports that independent control of the pore throat size parameters depends on using a single 2D plane structure with a deficiency of 3D space for *in vitro* cell tests. On this basis, studies have shown that when the pore throat size range is 50–100 μm, cells can form a membrane across the whole pore surface through morphological changes to block the pore, affecting the nutrient interaction and cell entry inside the pore structure, as shown in Figure 4A (Egles et al., 2013; Lei et al., 2021). However, when the pore throat size is > 200 μm, the cells no longer grow across the pores but show growth along the rod direction, and there is cell growth into the porous structure below its surface pores, as shown in Figure 4B (Liu et al., 2020; Sakthiabirami et al., 2021). This conclusion reflects the original implication of the pore throat size, which is a passage for cell entry. This size determines whether the cells can successfully enter the interior portion of the porous scaffold to perform their actual function (Deb et al., 2018). When the pore structure and pore throat size meet the above requirements, studies have shown that the smaller the pore throat size, the better the cell adhesion, proliferation, and differentiation for bone formation, as shown in Figure 4C (Yang et al., 2021). The main reason is that since the pore throat size reflects the angle, distance, and curvature between rods or walls in the pore structure, the specific mechanism by which the pore throat size causes differences in the cellular state may be related to differences in intracellular stress stimulation due to the morphology of cell adhesion and growth (Bershadsky et al., 2003; Rumpler et al., 2008; Bidan et al., 2013). On this basis, Fukuda et al. (2011) prepared cubic columnar canals of different sizes within the same scaffold to investigate the effect of pore throat size on the osteogenic effect of the scaffold pore structure *in vivo*. The results showed that the best internal bone tissue formation was achieved at a scaffold pore throat size of 500 μm and 5 mm from the end face, as shown in Figure 4D. It was also suggested that this might be related to the circulation of body fluids within the scaffold at different pore throat diameters. This conclusion further suggests that the pore throat size also interferes with the bone tissue formation within the porous material to some extent through the circulation of body fluids (Takahashi and Tabata, 2004; Van Bael et al., 2012). Considering the above studies, we believe that the main influence of pore throat size on bioengineered porous bone materials is mediated by controlling whether cells can enter the pore structure properly and circulating body fluids to influence cell adhesion and morphology.

### Function of pore size

Comparatively, *in vitro* cellular and *in vivo* animal studies on the effect of pore size on bone ingrowth and osteogenesis of porous scaffolds by pore structure shape or pore size are more complex than those related to pore throat size. In vitro cell studies, mostly after inoculating cells on the surface of different porous materials, the pore structure is adjusted to change the cell adhesion state and the intracellular stress, affecting the physiological activity of the cells (Diez-Escudero et al., 2020; Wu et al., 2022). This is the same mechanism by which the pore throat size interferes with cell adhesion and growth on the surface of porous scaffolds. However, Papaefstathiou et al. (2022) showed differences in early cell proliferation and final total cell number due to differences in surface morphology and surface area between the two groups of solid and porous disc samples. However, their normalized treatment did not show significant differences in ALP expression. In addition, Liang et al. (2022) reported no significant difference in the proliferation activity of cells inoculated on the surface of the materials, while there were differences in the pore size and pore morphology of porous materials. Wang et al. (2018b) carried out comprehensive cellular and animal experiments on porous scaffolds with four different pore structures. The results showed that the osteogenic effect within the porous scaffolds did not significantly correlate with cell adhesion, proliferation, and differentiation statuses. Therefore, we believe that because *in vitro* cellular experiments cannot simulate the complex growth environment and stress state of cells within the pore structure of porous materials, they are limited to studying the surface and 3D morphology of the pore structure of porous scaffolds. In addition, the results at the cellular level alone do not accurately represent the actual effects of porous materials after implantation *in vivo*. This is in general agreement with the conclusion reached by Karageorgiou and Kaplan, (2005) that the osteogenic effect of the pore structure of porous materials has two opposite tendencies *in vivo* and *in vitro*. However, with technological developments, some studies have been conducted on hydrogels to achieve a 3D co-culture system between porous scaffolds and cells. The results of these *in vitro* cell experiments will be closer to the actual *in vivo* state (Ji et al., 2020; Ma et al., 2021). Meanwhile, hydrodynamics is gradually becoming a new hotspot in the study of porous materials, where changes in pore shape, pore throat size, and pore size interfere with the permeability or fluid environment within the porous structure (Ma et al., 2019; Chao et al., 2021; Timercan et al., 2021), ultimately affecting changes in cell adhesion and proliferation (Markhoff et al., 2015; Liu et al., 2020). With the innovation of such experimental approaches, it will become a trend to further elucidate the mechanisms related to the cell growth condition within porous materials by constructing a bionic growth environment and applying specific mechanical stimuli to the cells within porous scaffolds *in vitro*.

**FIGURE 5:**
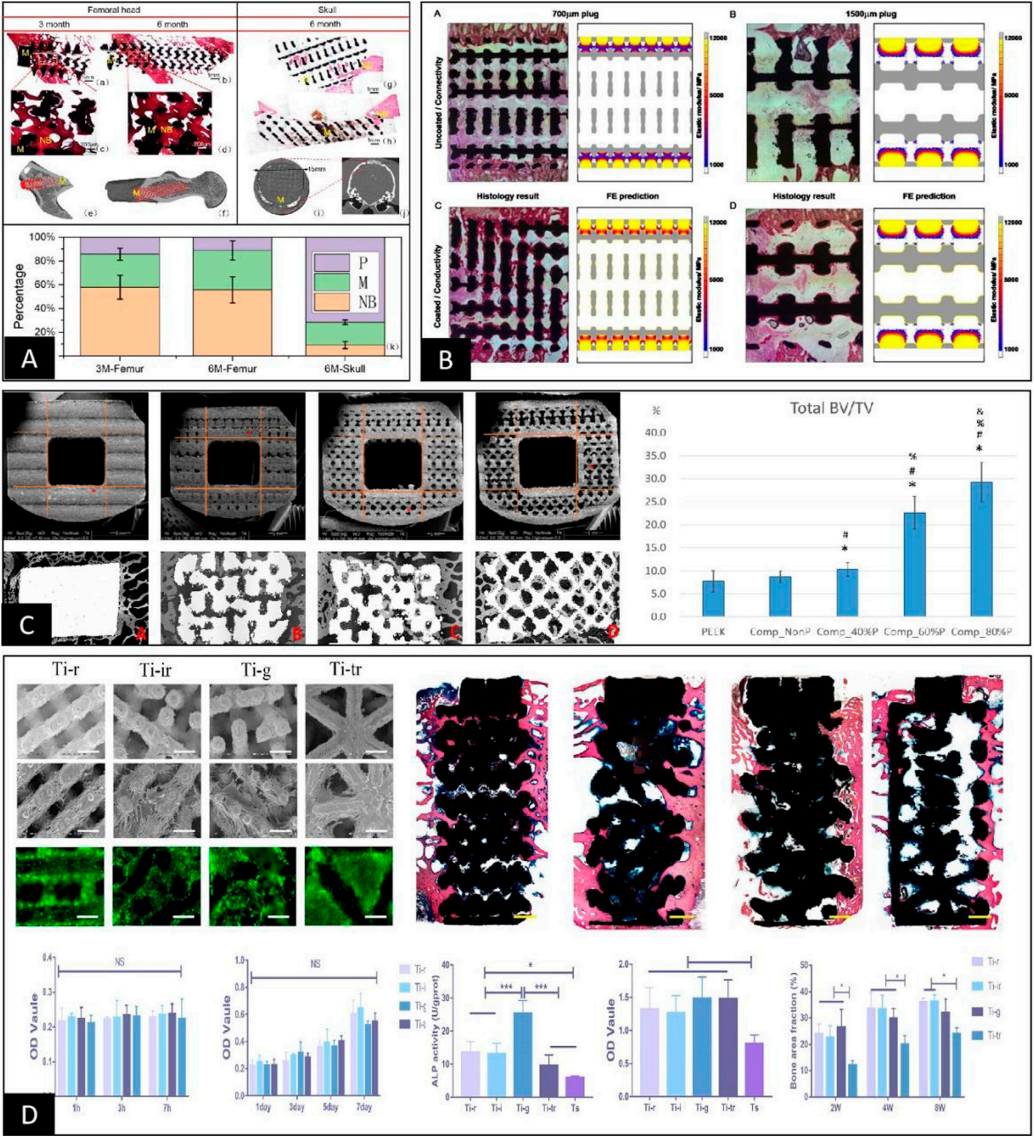
**(A)** Porous scaffolds with the same pore structure implanted in the femur and skull of the same animal were very different in the final osteogenic effect due to the difference in their local skeletal stresses (Pei et al., 2020). **(B)** Comparative analysis between finite element stress analysis and animal implantation experiments of porous scaffolds showed that the osteogenic region within the porous scaffold clearly correlated with stress distribution (Cheong et al., 2018). **(C)** The final post-implantation osteogenic effect of porous scaffolds with different elastic moduli constructed solely by rod diameter control was inversely correlated with the elastic modulus (Tsai et al., 2021). **(D)** After constructing porous scaffolds with different elastic moduli simply by different pore structures, their cellular assays showed no differences in cell adhesion, proliferation, and quantitative analysis of calcium nodules between the groups except for ALP activity. In contrast, animal tests showed differences in the final osteogenic effect between the groups, with no apparent correlation with the differences in elastic modulus and ALP activity of the porous scaffolds (Wang et al., 2018b).

At this stage, to make up for the shortcomings of *in vitro* cell experiments, researchers usually supplement *in vitro* animal experiments for further validation. However, the experimental results obtained through animal experiments are still highly controversial, mainly because parameters such as pore throat diameter, porosity, and elastic modulus change while adjusting the pore structure and pore size in 3D spaces (Zadpoor, 2015). First, even when porous scaffolds with the same material, preparation process, and pore structure are implanted in the skull and femur of the same animal, the effect of osteogenesis within the porous material is not uniform, mainly due to the different stress stimuli on the scaffold at different implantation sites, as shown in Figure 5A (Walsh et al., 2019; Pei et al., 2020). Furthermore, when a clear stress stimulus is missing at the implantation site, the osteogenic outcome is relatively poor, suggesting that osteogenesis within porous materials may be positively correlated with stress stimuli (Li et al., 2015; Taniguchi et al., 2016; Li et al., 2021; Lu et al., 2022b). The idea was also confirmed by Tsai et al. (2021) in an *in vivo* study by designing porous materials with the same pore size and different rod sizes. The results showed that the smaller the modulus of elasticity and the higher the porosity, the better the percentage of bone tissue volume within the porous material, as shown in Figure 5C. In addition, according to Cheong et al. (2018), the distribution range of osteogenesis within the porous scaffold observed by histomorphology in animal experiments was highly consistent with the stress distribution region within the pore structure under finite element analysis. This finding further suggests that the specific mechanism by which the pore structure affects osteogenesis may be related to the difference in the elastic modulus under the intervention of pore size, as shown in Figure 5B. However, Shah et al. (2016) showed that porous scaffolds using the same pore structure and different materials had essentially the same volume fraction of bone tissue at each site within both scaffolds after implantation and only differed in the integration of the bone‒metal interface. In addition, Wang et al. (2018b) undertook comprehensive animal experiments using porous scaffolds with four pore structures. The results showed no clear linear relationship between the osteogenic effect within the porous scaffold and parameters such as pore size, porosity, and elastic modulus, as shown in Figure 5D. Therefore, based on the results of different animal experimental osteogenesis analyses, we believe there is a clear correlation between the osteogenic effect within porous materials and the pore size. However, the specific mechanism of action is often related to the pore throat size, elastic modulus, and other related parameters that are not yet completely clear and still need further in-depth studies.

## Conclusion

We analyzed the origin and internal logic of different definition methods for the evolution of pore structure dimension characterization in bioengineered porous bone materials and proposed that it is more practical to characterize pore structure dimension by pore throat size and pore size together.

1)The pore throat size is the maximum cross-sectional diameter of the penetration channel of the cells into the interior portion of the pore structure. It is the maximum internal tangent circle diameter in the 2D plane at the surface of the pore structure. It can be calculated using SEM or Micro-CT or other methods by directly measuring the internal tangent circle diameter or cross-sectional equivalent circle diameter under 2D conditions by selecting a specific plane or cross-section.
2)The pore diameter is the maximum space diameter that can allow the cells to grow after entering the interior portion of the pore structure. It is the maximum internal tangential sphere diameter in the 3D spatial environment within the pore structure and can be measured by the rod or wall spacing equivalent to the pore diameter within the pore structure using SEM or Micro-CT or based on the reconstructed 3D model after Micro-CT scanning. The software simulation can be used to obtain its internal pore diameter distribution data. However, it is worth noting that the pore size distribution curve obtained in this way includes the pore throat size.

At the same time, based on the joint definition of pore throat size and pore size, the specific functions and mechanisms of their respective roles in bioengineered porous bone materials were analyzed. The results showed that both pore throat size and pore size could affect the cell growth state and the final osteogenesis in porous scaffolds in different ways.

1)The pore throat size, which is the size of the channel that characterizes the internal access of cells to the pore structure, directly determines whether the cells can enter the pore structure smoothly. It also determines the specific state of the circulation of body fluids between the internal and external shelf tissues of the pore structure, which influences the specific process of osteogenesis within the pore structure in the form of nutrient supply. At the same time, the morphology and size of the pore throat, as a direct morphological structure perceived by cells adhering to the surface of the pore structure, can also affect the specific functions of cell proliferation and differentiation by changing the cell adhesion status. However, this conclusion is limited to the cellular level and has not been confirmed in animal experiments.
2)As a characterization of the size of the space in which cells can grow within the pore structure, the pore size also represents the pore structure dimensions. The function of pore size at the cellular level is similar to that of pore throat size in that it changes the cellular adhesion state through morphology and size, affecting the specific functions of cell proliferation and differentiation. However, the conclusions of such cellular-level studies are not fully consistent with the results of actual porous scaffold implantation *in vivo*, mainly because the cellular experiments lack the complex physiological environment and the mechanical stimuli *in vivo*. The function of the pore size *in vivo* is to co-intervene with parameters such as pore shape and rod diameter in the elastic modulus of the material to change the distribution of stress stimuli within the porous scaffold and influence the osteogenic state within the pore structure.

Currently, bioengineered porous bone tissue materials and their related products are initially applied in the first line of clinical practice. However, there are still various problems, such as intraoperative sinking, non-fusion of bone graft, pseudo-joint formation, postoperative implant infection, *etc.* The root cause of these problems is the lack of in-depth research on the pore structure and the inability to clarify the specific mechanisms of osteogenesis, vascularization, and fibrogenesis within the pore structure of porous materials, which cannot be precisely controlled and regulated. This paper proposed the characterization of pore structure dimension by pore size and pore throat size by reviewing, summarizing, and unifying the specific definition and measurement methods of pore size and pore throat size. On this basis, the possible roles and mechanisms of specific parameters of pore structure dimension that influence osteogenesis within porous materials were proposed to provide further theoretical references for the subsequent in-depth studies of pore structure.
